# Epithelial Aryl Hydrocarbon Receptor Protects From Mucus Production by Inhibiting ROS-Triggered NLRP3 Inflammasome in Asthma

**DOI:** 10.3389/fimmu.2021.767508

**Published:** 2021-11-15

**Authors:** Xinyue Hu, Yingchun Shen, Yilin Zhao, Ji Wang, Xin Zhang, Wei Tu, William Kaufman, Juntao Feng, Peisong Gao

**Affiliations:** ^1^ Division of Allergy and Clinical Immunology, Johns Hopkins University School of Medicine, Baltimore, MD, United States; ^2^ Department of Respiratory Medicine, Xiangya Hospital, Central South University, Changsha, China; ^3^ Department of Respiratory Medicine, Xijing Hospital, The Fourth Military Medical University, Xi’an, China; ^4^ Department of Respiratory and Critical Care Medicine, West China Hospital, Sichuan University, Chengdu, China; ^5^ Laboratory of Pulmonary Immunology and Inflammation, Frontiers Science Center for Disease-Related Molecular Network, Sichuan University, Chengdu, China; ^6^ Department of Integrated Traditional Chinese and Western Medicine, West China Hospital, Sichuan University, Chengdu, China; ^7^ Department of Respirology & Allergy, Third Affiliated Hospital of Shenzhen University, Shenzhen, China

**Keywords:** asthma, cockroach allergen, aryl hydrocarbon receptor, ROS, inflammasome

## Abstract

**Background:**

Despite long-standing recognition in the significance of mucus overproduction in asthma, its etiology remains poorly understood. Muc5ac is a secretory mucin that has been associated with reduced pulmonary function and asthma exacerbations.

**Objectives:**

We sought to investigate the immunological pathway that controls Muc5ac expression and allergic airway inflammation in asthma.

**Methods:**

Cockroach allergen-induced Muc5ac expression and aryl hydrocarbon receptor (AhR) signaling activation was examined in the human bronchial epithelial cells (HBECs) and mouse model of asthma. AhR regulation of Muc5ac expression, mitochondrial ROS (Mito-ROS) generation, and NLRP3 inflammasome was determined by AhR knockdown, the antagonist CH223191, and AhR^-/-^ mice. The role of NLRP3 inflammasome in Muc5ac expression and airway inflammation was also investigated.

**Results:**

Cockroach allergen induced Muc5ac overexpression in HBECs and airways of asthma mouse model. Increased expression of AhR and its downstream genes CYP1A1 and CYP1B1 was also observed. Mice with AhR deletion showed increased allergic airway inflammation and MUC5AC expression. Moreover, cockroach allergen induced epithelial NLRP3 inflammasome activation (e.g., NLRP3, Caspase-1, and IL-1β), which was enhanced by AhR knockdown or the antagonist CH223191. Furthermore, AhR deletion in HBECs led to enhanced ROS generation, particularly Mito-ROS, and inhibition of ROS or Mito-ROS subsequently suppressed the inflammasome activation. Importantly, inhibition of the inflammasome with MCC950, a NLRP3-specifc inhibitor, attenuated allergic airway inflammation and Muc5ac expression. IL-1β generated by the activated inflammasomes mediated cockroach allergen-induced Muc5ac expression in HBECs.

**Conclusions:**

These results reveal a previously unidentified functional axis of AhR-ROS-NLRP3 inflammasome in regulating Muc5ac expression and airway inflammation.

## Introduction

Asthma is a common chronic disease characterized by inflammation and bronchial obstruction. Mucus overproduction has been considered as one of the major contributors to the asthma morbidity and mortality (1). Particularly, airway mucus plugging has been associated with impaired pulmonary function and is the most common clinical feature in patients with fatal asthma (2–6). Muc5ac secreted by the goblet cells within epithelium is the predominant mucin glycoprotein implicated in fatal asthmatic airway mucus plugging (1, 3, 7). Muc5ac over-expression has been associated with the development of airway mucus plugging, allergic airway hyper-reactivity, and progressive loss of lung function, and asthma exacerbations (7–10). A very recent finding from a multicenter, observational study suggests that increased Muc5ac concentration in induced sputum was a major driver in COPD initiation, progression, exacerbation risk, and overall pathogenesis (11). Furthermore, genetic studies have provided evidence that genetic variations in the Muc5ac region of chromosome 11 were associated with asthma and contribute to Muc5ac expression that may underlie propensity to mucus plugging during asthma exacerbations (12). However, despite overwhelming evidence of a pathogenic role for Muc5ac in asthma, the immunological pathway that controls Muc5ac overexpression remains poorly understood.

Aryl hydrocarbon receptor (AhR) is a unique cellular ‘‘chemical sensor’’ and a ligand-activated transcription factor that can interact with the endogenous and exogenous organic molecules, including tryptophan metabolites, microbial metabolites, and environmental pollutants (13–18). Upon ligand binding, AhR translocates from the cytosol to the nucleus and binds to a dioxin response element (DRE) of several xenobiotic-metabolizing enzymes (e.g., *cytochrome P450, CYP1A1, CYP1B1*) that are required for detoxification (19–21). Recently, AhR has been associated with asthma (22–26) and shown to mediate environmental pollutant-enhanced allergic lung inflammation (23, 27–29), ROS-dependent degranulation and activation of mast cells (30, 31). We have recently demonstrated that benzo(a)pyrene (BaP, a ubiquitous air pollutant) co-exposure with Der f 1 (a common allergen to human) induced the activation of AhR signaling, which subsequently regulated the co-exposure-induced airway epithelial cell oxidative stress and cytokine release (32, 33). Importantly, several studies have suggested that the BaP-induced activation of AhR signaling regulates Muc5ac overexpression in airway epithelial cells (34, 35). However, the role of the allergen-activated AhR signaling pathway in regulating Muc5ac overexpression and its precise mechanisms remains poorly understood.

NLRP3 inflammasome is an intracellular sensor that detects environmental irritants and endogenous danger signals and has been suggested as an important therapeutic target for human metabolic and autoinflammatory diseases (36–39). It is composed of the inflammation sensor protein NLRP3, apoptotic speck protein containing a caspase recruitment domain (ASC), and the effector protein caspase-1 (40). NLRP3 inflammasome activation triggers the conversion of procaspase-1 to caspase-1, consequently promoting the maturation of IL-1β and IL-18, which contribute to increased airway inflammation (41, 42). While NLRP3 inflammasome has been considered to be critical in the clearance of pathogens in the airways, study suggests that environmental allergen and irritant-induced persistent NLRP3 inflammasome activation plays a strong role in the exacerbation of asthma features (43). Intriguingly, NLRP3 inflammasome has been shown to induce Muc5ac production in airway epithelial cells (37, 44, 45) and play a role in allergic airway inflammation (42, 46–49). Thus, it seems plausible that the NLRP3 inflammasome is a central player in mediating the AhR signaling pathway-regulated Muc5ac expression. Furthermore, studies have indicated that mitochondrial ROS are required for NLRP3 inflammasome activation in bronchial epithelial cells (39, 46, 50). These findings, therefore, raise the possibility that allergen-activated AhR signaling controls mitochondrial ROS generation that subsequently activates NLRP3 inflammasome and induces Muc5ac expression in asthma.

In the present study, we report that cockroach allergen exposure can induce Muc5ac expression, activate AhR signaling pathway, and trigger NLRP3 inflammasome activation by both *in vitro* and *in vivo* analyses. Further studies address that epithelial AhR is required in protecting against cockroach allergen-induced Muc5ac expression and NLRP3 inflammasome activation. Mechanistically, we provide evidence that AhR regulates NLRP3 inflammasome activation through controlling cockroach allergen-induced ROS, particularly mitochondrial ROS generation. Importantly, inhibition of NLRP3 inflammasome protects against allergen-airway inflammation and Muc5ac expression. Thus, the functional axis of AhR-ROS-NLRP3 inflammasome could lead to new therapeutic perspectives for asthma.

## Methods

### Mice

Both C57BL/6J and *AhR deficient* (*AhR^-/-^*) mice on the C57BL/6 background purchased from the Jackson Laboratories (Bar Harbor, Me) were used in this study. All mice were used at 6-8 weeks of age, and all experiments used age- and sex-matched controls. All animals were maintained under specific pathogen-free conditions in the animal facility of the Johns Hopkins University School of Medicine. The animal care and experiments were performed in compliance with the institutional and US National Institutes of Health guidelines and were reviewed and approved by the Johns Hopkins University Animal Care and Use Committee.

### Cockroach Allergen-Induced Asthma Mouse Model

**Figure 1:**
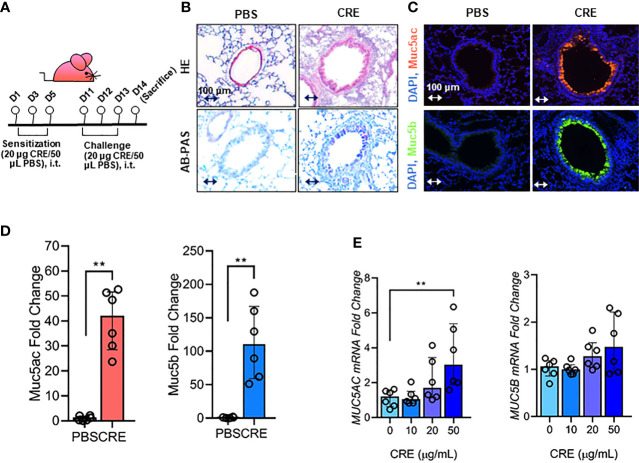
Cockroach allergen exposure can induce airway inflammation and mucus production. **(A)** Experimental scheme for cockroach allergen-induced mouse model of asthma. **(B)** Histological examination of mouse paraffin lung sections stained with hematoxylin and eosin (H&E, upper panel) and Alcian Blue periodic acid-Schiff (AB-PAS, lower panel). **(C)** Representative immunofluorescence images of Muc5ac (upper panel) and Muc5B (lower panel) in the airways of cockroach allergen-induced mouse model. **(D)** Quantitative analysis of [**(C)**, n = 6]. **(E)** RT-PCR analysis of Muc5ac and Muc5B in HBECs treated with cockroach extract (CRE) at different concentrations for 24 h (n = 6). Data represent as medians with interquartile (IQR). **P < 0.01.

Cockroach allergen-induced asthma mouse model was established with the protocol revised from our previous work (51) and illustrated in **Figure 1A**. Mice were sacrificed and samples were collected on the next day after the last allergen challenge. In a separate experiment, mice were treated with MCC950 (10 mg/kg, Selleckchem, USA) dissolved in sterile distilled water by intraperitoneal administration 1 hour before every single allergen challenge (52).

### Analysis of Lung Inflammation

Mouse lungs were perfused with 10 mL of ice-cold PBS injected into the right ventricle followed by excision and fixation in 4% formalin and embedding in paraffin. Sections (4 µm) were prepared from paraffin-embedded lungs and then subjected to hematoxylin and eosin (H&E) and Alcian Blue periodic acid-Schiff (AB-PAS) staining to evaluate general morphology and mucus production as previously described (53).

### Flow Cytometry Analysis

For analysis of bronchial lavage (BAL) fluids, total cells in BAL fluid and cellular differential percentages were determined by flow cytometry on an Accuri C6 Plus Flow Cytometer (BD Biosystems), and the data were analyzed with FlowJo software (Treestar). Eosinophils are characterized as SSC^hi^SiglecF^+^Mac-3^-^, alveolar macrophages as SSC^hi^SiglecF^+^Mac-3^+^, granulocytes as SSC^hi^ Gr-1^+^, and lymphocytes as FSC^lo^/SSC^lo^ and CD3^+^ cells (54).

### Cell Culture and Treatment

Human bronchial epithelial cells (HBEC3-KT, ATCC CRL-4051), an immortalized cell line, were cultured in Ham’s F-12K (Kaighn’s) medium supplemented with 10% v/v fetal bovine serum (FBS) and 1% penicillin‐streptomycin. Cockroach extract (CRE, Greer Laboratory) was used to treat HBECs. In separate experiments, HBECs were pre-treated with AhR antagonist CH-223191 (Sigma, C8124, 10 µM), antioxidant NAC (Sigma, A9165, 10mM), MitoTEMPO (sigma, SML0737, 100 µM), or MCC950 (Selleckchem, S7809, 10 µM) or IL-1β neutralizing antibody (R&D, MAB601, 2 µg/ml), or vehicle controls 1 hour before CRE treatment.

### Assessment of Intracellular ROS Production

Intracellular ROS were detected by Flow cytometry with the general oxidative sensitive fluorescent dyes CM-H_2_DCFDA (ThermoFisher) as previously reported (55).

### Transfection of siRNA

AhR knockdown in HBECs was accomplished by using a pre-designed Mission siRNA pair (Sigma-Aldrich, SASI_Hs01_00140198). siRNA transfection was performed with Lipofectamine™ RNAiMAX (ThermoFisher) following the manufacture’s instruction, while control cells received negative control siRNA. Cells were cultured for 48 hours and transfection efficiency was evaluated by quantitative real-time PCR and western blot.

### RNA Isolation and Quantitative Real-Time PCR Analysis

Total RNA from lung tissues or HBECs were isolated using the Monarch Total RNA Miniprep Kit (New England BioLabs) and cDNAs were synthesized with the High-Capacity cDNA Reverse Transcription Kit (ThermoFisher). Quantitative real-time PCR (RT-PCR) analysis was performed using Power SYBR Green PCR Master Mix (ThermoFisher) on an ABI Prism 7300 detection system. Data were analyzed using the 2^-ΔΔCT^ method relative to the housekeeping gene Actin (56). Primer sequences are provided in the Online Repository (See **Table E1**).

### Immunofluorescence Staining

Immunofluorescence staining was performed as previously reported (53). Antibody information is provided in **Table E2**. The intracellular superoxide in lung tissues was detected using dihydroethidium (DHE) (ThermoFisher). Intracellular ROS in HBECs were detected using the mitochondria-specific oxidative sensitive fluorescent dyes MitoSOX Red and Mito Tracker (ThermoFisher) as previously reported (53). The sections were mounted with the Fluoromount Mounting medium (Sigma) and observed by a NIKON ECLIPSE Ti-U microscope equipped with DS-Fi2 camera (NIKON, USA). Fluorescence signals in tissue sections were quantified using ImageJ v1.50e (NIH) in four different high-power fields from each lung section and presented as mean fluorescence intensity per square micrometer.

### Enzyme-Linked Immunosorbent Assay

Levels of cytokines in cell-free BAL fluid or supernatant were quantified by using the Ready-Set-Go! ELISA sets (ThermoFisher). Cockroach allergen-specific IgE and IgG1 serum levels were analyzed by ELISA as described previously (53). Absorption at 450 nm was measured with an iMark Microplate Absorbance Reader (BioRad).

### Western Blotting

Tissues or cells were lysed with RIPA buffer containing protease and phosphatase inhibitor cocktails (Sigma-Aldrich). Protein concentrations were measured by using BCA kit (Thermo Fisher Scientific). An equal amount of total protein (20-50 µg) was loaded onto a 4%-12% Tris-Glycine Gel in NuPAGE MOPS SDS Running Buffer (Thermo Fisher Scientific) and then transferred using the iBlot2 NC Stack System (ThermoFisher). The membranes were blocked in 5% non-fat milk in TBST for 1 h at room temperature and then probed with primary antibodies overnight at 4°C. Species-appropriate secondary antibodies conjugated to IRDye 680RD or IRdye 800CW (LI-COR Biosciences) were used according to the manufacturer’s instructions. Detection was performed using iBright 1500 Imaging Systems and fluorescent intensity was quantified using the system built in IBright Analysis Software (Thermo Fisher Scientific).

### Statistical Analysis

Data were analyzed using GraphPad Prism version 8.2.1 (GraphPad Software, La Jolla, CAUSA). For data with >6 samples/group, comparison of two groups was performed by student’s two-tailed unpaired t-test. Comparison of more than two groups was performed by ordinary one-way ANOVA followed by Tukey’s post-hoc test. Data are presented as means ± SEM. For those with ≤6 samples/group, comparison of two groups was performed by Mann Whitney test, and comparison of more than two groups was performed by Kruskal-Wallis followed by Dunn’s post-hoc test. Data are presented as medians with interquartile (IQR). *P* < 0.05 was considered statistically significant.

## Results

### Cockroach Allergen Exposure Induces Airway Mucus Production

Our first objective was to determine whether cockroach allergen can induce mucus production. Using a well-established cockroach allergen-induced asthma mouse model as illustrated in **Figure 1A**, we examined cockroach allergen-induced lung inflammation and mucus production by using H&E and AB-PAS staining, respectively (**Figure 1B**). The AB-PAS technique is the most sensitive method for detection of all mucins. Consistent with our previous findings (55, 57), cockroach allergen induced increased lung inflammation and mucus production compared to PBS treatment. The increased mucus production was further supported by immunofluorescence staining. Both Muc5ac and Muc5b (8), two major components of the mucus, were highly expressed in the airways of cockroach allergen-induced mouse model (**Figures 1C, D**). Expression of Muc5ac and Muc5b was confirmed in HBECs in response to cockroach extract (CRE). A dose-dependent response was observed for Muc5ac, but not Muc5b, in HBECs after treatment with CRE (**Figure 1E**). These findings suggest that cockroach allergen exposure can induce airway inflammation and Muc5ac overexpression.

### Cockroach Allergen Exposure Induces Activation of AhR Signaling Pathway

**Figure 2:**
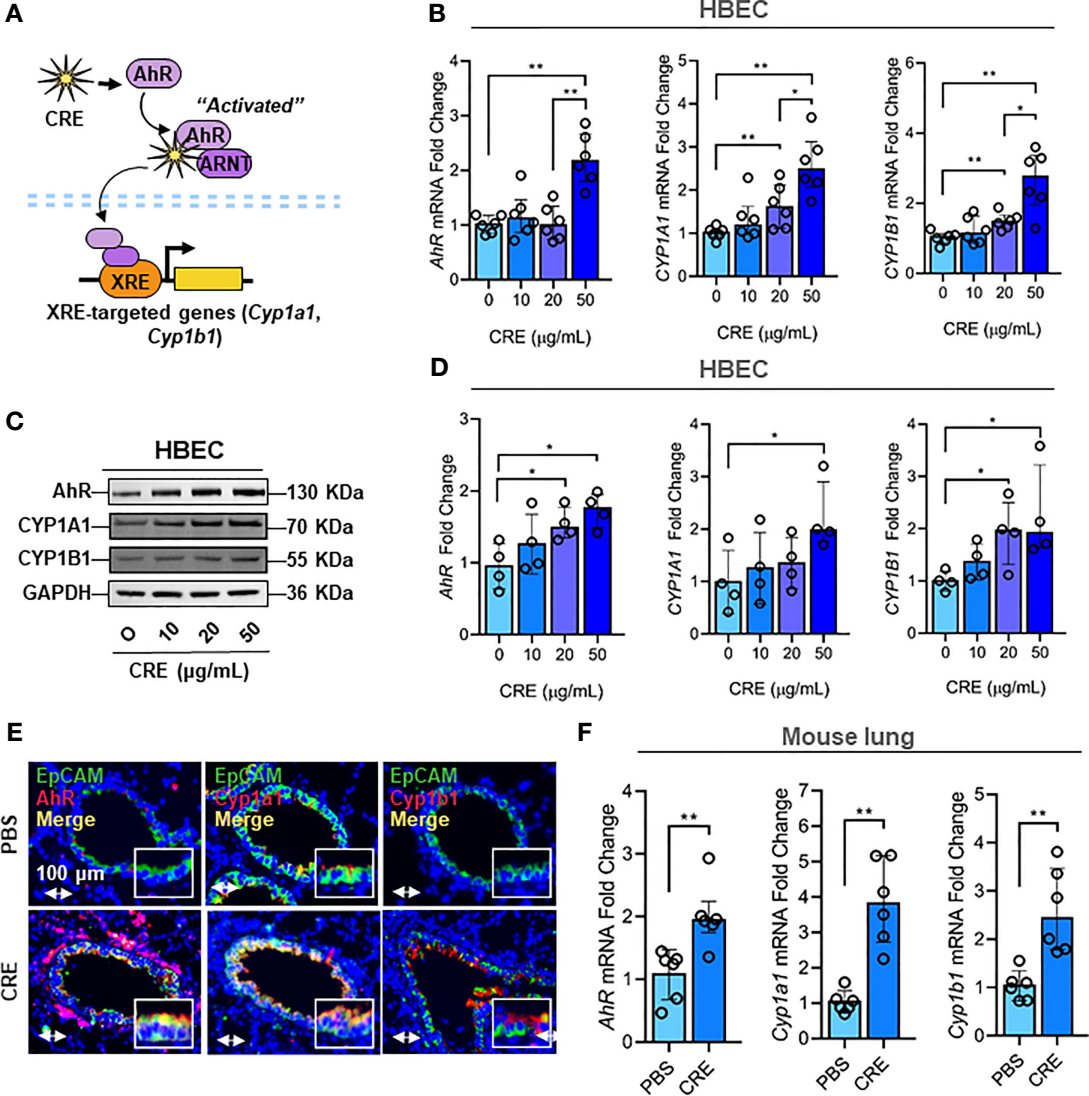
Cockroach allergen exposure induces activation of AhR signaling pathway. **(A)** Scheme of cockroach allergen-induced the activation of AhR signaling. **(B, C)** expression of AhR, CYP1A1 and CYP1B1 in cockroach extract (CRE)-treated HBECs at different concentrations for 24 h (n = 6) as detected by RT-PCR [**(B)** n = 6] and western blot **(C)**. GAPDH was detected as the loading control. **(D)** Quantitative analysis of western blots [**(C)**, n = 4]. **(E)** Representative immunofluorescence images of AhR, Cyp1a1 and Cyp1b1 (red) in the airway epithelial cells (EpCAM, green) of cockroach allergen-induced mouse model. **(F)** Quantitative analysis of florescent signal [**(E)**, n = 6]. Data represent as medians with interquartile (IQR). *P < 0.05, **P < 0.01.

Next, we investigated the molecular mechanisms underlying the cockroach allergen-induced mucus production with a special focus on AhR signaling pathway. Our previous study has demonstrated that AhR protects lungs from allergen-induced inflammation by modulating mesenchymal stem cell recruitment and immune-suppressive activity (58). Thus, we examined whether cockroach allergen can induce AhR activation specifically in the airway epithelial cells as determined by the expression of AhR and its downstream genes CYP1A1 and CYP1B1 in HBECs (**Figure 2A**). As expected, RT-PCR analysis indicated that CRE induced the expression of AhR, CYP1A1, and CYP1B1 in HBECs in a dose-dependent manner (**Figure 2B**). The increased expression of AhR, CYP1A1, and CRP1B1 was further confirmed by western blot analysis (**Figures 2C, D**). Consistently, significantly increased expression was observed for AhR, Cyp1a1, and Cyp1b1 in the airway epithelial cells of CRE-treated mouse model as detected by co-immunofluorescent staining (**Figures 2E, F**). These data reveal that cockroach allergen can induce epithelial AhR signaling activation.

### Cockroach Allergen-Activated AhR Protects Airway Inflammation and Muc5ac Production

**Figure 3:**
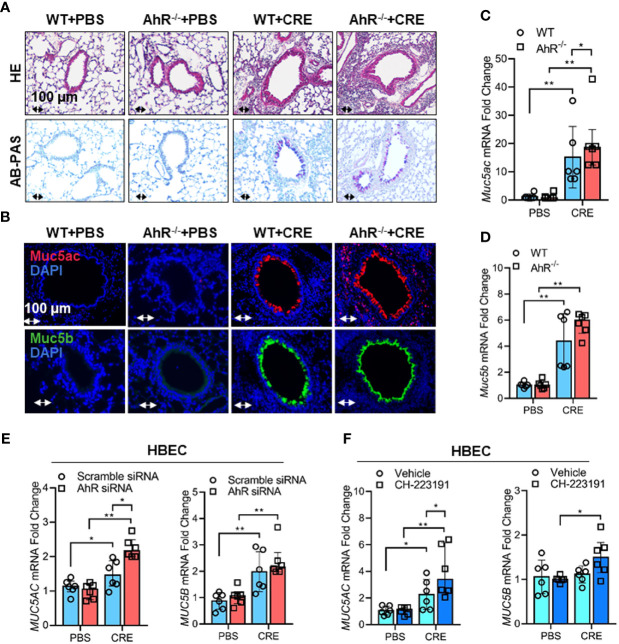
Cockroach allergen-activated AhR protects airway inflammation and Muc5ac production. **(A)** Histological examination of mouse paraffin lung sections stained with hematoxylin and eosin (H&E, upper panel) and Alcian Blue periodic acid-Schiff (AB-PAS, lower panel). **(B)** Representative immunofluorescence images of Muc5ac (red) and Muc5B (green) in the lung tissues of cockroach allergen-induced mouse model with wild-type (WT) and AhR^-/-^ mice (n = 6). **(C, D)**, RT-PCR analysis of Muc5ac **(C)** and Muc5B **(D)** in the lung tissues of cockroach allergen-induced mouse model (n = 6). **(E)** RT-PCR analysis of Muc5ac and Muc5B in cockroach extract (CRE, 50 µg/ml)-treated HBECs with or without AhR knockdown (n=6). **(F)** RT-PCR analysis of Muc5ac and Muc5B in CRE-treated HBECs with or without CH223191 (10 µM, n = 6). Data represent as medians with interquartile (IQR). *P < 0.05, **P < 0.01.

Given that cockroach allergen exposure can induce AhR expression and signaling activation in the airway epithelial cells, following studies investigated whether AhR plays a role in regulating cockroach allergen-induced mucus production. By using our asthma mouse model with AhR deficient (AhR^-/-^) mice, we found that cockroach allergen-induced increased airway inflammation (H&E, upper panel) and mucus production (AB-PAS, lower panel) were enhanced in AhR^-/-^ mice compared with WT mice (**Figure 3A**). The same pattern was observed for total and eosinophil cell counts in BAL fluids (**Figure E1A**), serum levels of CRE-specific IgE and IgG1 (**Figure E1B**), and Th2 cytokines in BAL samples (**Figure E1C**). When Muc5ac and Muc5b expression were specifically analyzed in the mouse model, we found that they were highly expressed in the airways of CRE-treated mouse model as determined by immunofluorescence staining, and the increased Muc5ac, but not Muc5b, was further strengthened in CRE-treated AhR^-/-^ mice relative to WT mice (**Figure 3B**). This finding was further confirmed in lung tissues by RT-PCR analysis. Lung tissues from CRE-treated AhR^-/-^ mice showed increased expression of Muc5ac as compared to those from WT mice (**Figure 3C**), but no change was observed for Muc5b (**Figure 3D**). To further confirm whether AhR in the airway epithelial cells plays a role in regulating Muc5AC and Muc5B expression, AhR in HBECs was knocked down by siRNA, which was confirmed by RT-PCR (**Figure E2A**) and western blot (**Figure E2B**). Indeed, the CRE-induced Muc5ac expression in HBECs was further promoted in HBECs with AhR knockdown (**Figure 3E**). No major change was noted for MUC5B. Similar results were found in HBECs pretreated with or without AhR inhibitor CH223191 (**Figure 3F**). Our findings indicate that AhR protects against cockroach allergen-induced airway inflammation and mucus production.

### AhR Protects Against Cockroach Allergen-Induced NLRP3 Inflammasome in Airway Epithelial Cells

**Figure 4:**
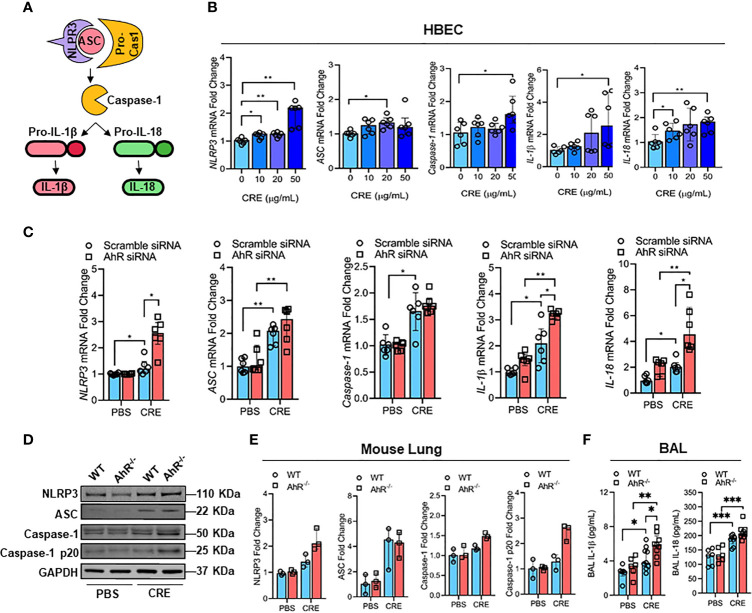
AhR protects against cockroach allergen-induced NLRP3 inflammasome in airway epithelial cells**. (A)** Scheme for NLRP3 inflammasome activation. **(B)** Cockroach allergen-induced NLRP3 inflammasome activation as detected by RT-PCR (n = 6). **(C)** Expression of NLRP3 inflammasome components in cockroach extract (CRE, 50 µg/ml)-treated HBECs with or without AhR knockdown as detected by RT-PCR (n = 6). **(D)** Western blot analysis of NLRP3 inflammasome components in the lung tissues of cockroach allergen-induced mouse model with wild-type (WT) and AhR^-/-^ mice. **(E)** Quantitative analysis of western blots **(E)**, n = 3]. **(F)** Levels of IL-1β and IL-18 in the BAL fluids of cockroach allergen-induced mouse model with wild-type (WT) and AhR^-/-^ mice (n = 6-9). Data represent as medians with interquartile (IQR) **(B, C, E)** or means ± SEM **(F)**. *P < 0.05, **P < 0.01, ***P < 0.001.

It was documented that NLRP3 inflammasome activation triggers the conversion of procaspase-1 to caspase-1 and consequently promotes maturation of IL-1β and IL-18, thereby leading to an increased airway inflammation (41, 42) (**Figure 4A**). However, it remains uncertain whether cockroach allergen can induce NLRP3 inflammasome activation and AhR is required in cockroach allergen-induced NLRP3 inflammasome activation. As expected, RT-PCR analyses demonstrated increased expression of NLRP3 inflammasome components NLRP3, ASC, Caspase-1, IL-1β, and IL-18 in HBECs after exposure to different doses of CRE (**Figure 4B**). Next, we examined whether AhR regulates cockroach allergen-induced NLRP3 inflammasome activation in HBECs. Compared to WT HBECs, HBECs with AhR knockdown showed an increase in the expression of NLRP3 and pro-inflammatory cytokines IL-1β and IL-18 after treatment with CRE (**Figure 4C**). While the quantification analyses didn’t reach statistical significance, western blot analysis demonstrated similar trends for the enhanced NLRP3 inflammasome activation in HBECs with AhR knockdown (**Figures E3A, B**). Similar results were also noted for the enhanced NLRP3 inflammasome activation in the lung tissues from CRE-treated AhR^-/-^ mice when compared with those from WT mice (**Figures 4D, E**). Additionally, both IL-1β and IL-18 were measured by ELISA (**Figure 4F**), IL-1β, but not IL-18, showed a significant increase in the BAL fluids of CRE-treated AhR^-/-^ mice relative to WT mice. Collectively, our results suggest that cockroach allergen can induce NLRP3 inflammasome activation and epithelial AhR protects against cockroach allergen-induced NLRP3 inflammasome.

### AhR Regulates Cockroach Allergen-Induced Airway Epithelial Mitochondrial ROS Generation

**Figure 5:**
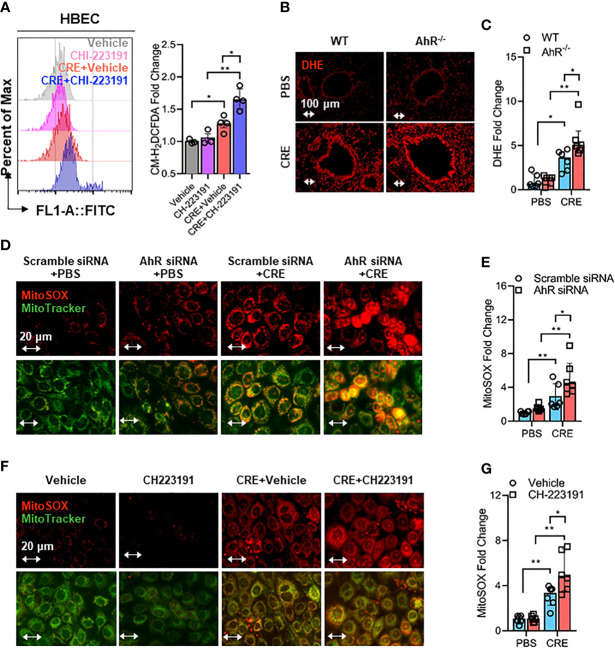
Epithelial AhR prevents cockroach allergen-induced airway epithelial mitochondrial ROS generation. **(A)** Intracellular ROS production in HBECs were detected by flow cytometry analysis with CM-H2DCFDA (n = 4). **(B)** Representative immunofluorescence images of ROS expression with dihydroethidium (DHE) in the lung tissues of cockroach allergen-induced mouse model with wild-type (WT) and AhR^-/-^ mice. **(C)** Quantitative analysis of florescent signals [**(B)**, n = 6]. **(D)** Representative immunofluorescence images of mitochondrial ROS expression with MitoSOX in cockroach extract (CRE, 50 µg/ml)-treated HBECs with or without AhR knockdown. **(E)** Quantitative analysis of florescent signals [**(D)**, n = 6]. **(F)** Representative immunofluorescence images of mitochondrial ROS expression with MitoSOX in cockroach extract (CRE, 50 µg/ml)-treated HBECs with or without CH223191 (10 µM). **(G)** Quantitative analysis of florescent signals [**(F)**, n = 6]. Data represent as medians with interquartile (IQR). *P < 0.05, **P < 0.01.

Mitochondrial ROS are crucial for NLRP3 inflammasome activation in bronchial epithelial cells (39, 46, 50), raising the possibility that AhR signaling controls NLRP3 inflammasome activation through modulating mitochondrial ROS generation. Thus, we determined whether AhR regulates cockroach allergen-induced ROS generation, particularly Mitochondrial ROS production. Increased intracellular ROS were observed in CRE-treated HBECs as evaluated by flow cytometry with CM-H2DCFDA (**Figure 5A**). The augmented intracellular ROS were further potentiated when the CRE-treated HBECs were pre-treated with AhR antagonist CH223191. This finding was supported by the *in vivo* analyses of ROS production in the lung tissues of CRE-treated mice by dihydroethidium (DHE) immunostaining (**Figures 5B, C**). Lung tissues from CRE-treated AhR^-/-^ mice showed a significant increase in ROS production when compared with those from WT mice. Similar results were observed for mitochondrial ROS production. Compared to untreated HBECs, CRE-treated HBECs had increased mitochondrial ROS as determined by co-immunostaining for MitoTracker green and MitoSOX, a fluorescent mitochondrial ROS reporter dye (**Figures 5D, E**). Notably, the increased mitochondrial ROS were markedly enhanced in HBECs with AhR knockdown. The findings were supported by using AhR antagonist CH223191 (**Figures 5F, G**). Taken together, our results support the notion that AhR protects against cockroach allergen-induced epithelial ROS production, particularly mitochondrial ROS production.

### ROS Are Required in Cockroach Allergen-Induced NLRP3 Inflammasome Activation

**Figure 6:**
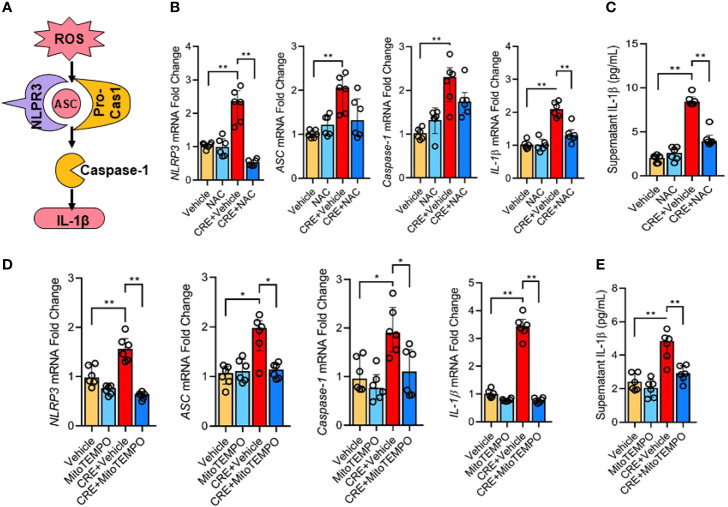
ROS is required in cockroach allergen-induced NLRP3 inflammasome activation. **(A)** Scheme for ROS-induced NLRP3 inflammasome activation. **(B)** Expression of NLRP3 inflammasome components) in CRE-treated HBECs in the presence or absence of NAC as detected by RT-PCR (n = 6). **(C)** Levels of IL-1β in supernatant of CRE-treated HBECs in the presence or absence of NAC (n = 6). **(D)** Expression of NLRP3 inflammasome activation components in CRE-treated mice in the presence or absence of Mito TEMPO as detected by RT-PCR (n = 6). **(E)** Levels of IL-1β in supernatant of CRE-treated HBECs in the presence or absence of Mito TEMPO (n=6). Data represent as medians with interquartile (IQR). *P < 0.05, **P < 0.01.

Next, we investigated whether the AhR-regulated ROS production is required for the cockroach allergen-induced NLRP3 inflammasome activation as illustrated in **Figure 6A**. Consistent with our previous observation, CRE treatment led to an increased expression of NLPR3, ASC, Caspase-1, and IL-1β as detected by RT-PCR analysis in HBECs (**Figure 6B**). Intriguingly, the increased NLPR3 and IL-1β were markedly blocked in pretreated HBECs with antioxidant N-acetyl cysteine (NAC). The result was also observed for IL-1β in supernatants of HBECs as measured by ELISA (**Figure 6C**). These findings were further supported by western blot analysis (**Figures E4A, B**) that showed inhibition for the increased expression of NLPR3, caspase-1, caspas-1 p20, and IL-1β by NAC treatment. Similar results were also observed in HBECs pretreated with mitochondrial-targeted antioxidant MitoTEMPO (**Figure 6D**). The CRE-induced increased expression of NLPR3, ASC, caspase-1, and IL-1β was significantly suppressed by MitoTEMPO treatment. In addition., the increased IL-1β in supernatants was noted in pre-treated HBECs with MitoTEMPO (**Figure 6E**). These data reveal that ROS are required for cockroach allergen-induced NLRP3 inflammasome activation.

### Inhibition of NLRP3 Inflammasome Alleviates Cockroach Allergen-Induced Airway Inflammation

**Figure 7:**
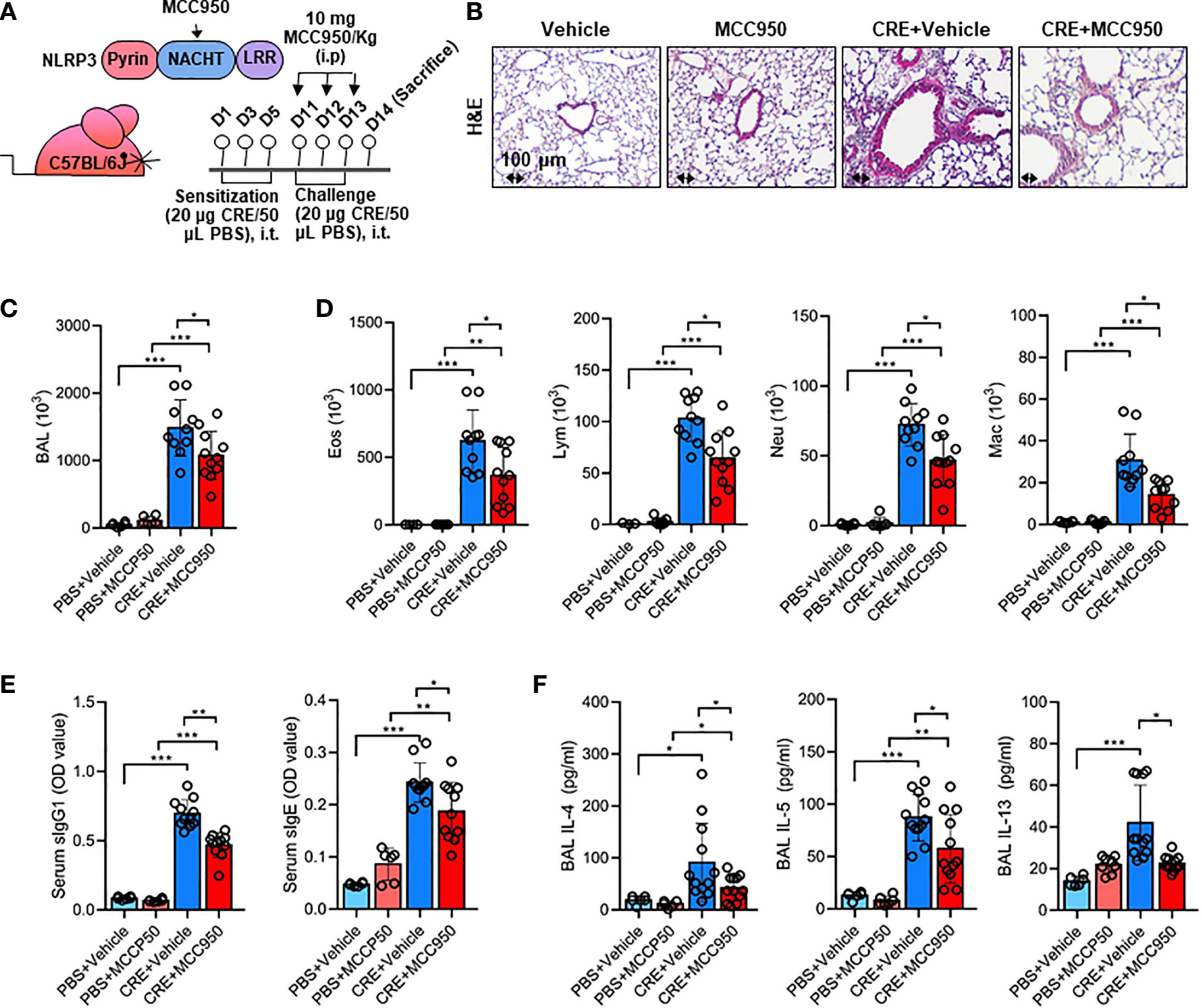
Inhibition of NLRP3 inflammasome alleviates cockroach allergen-induced airway inflammation. **(A)** Experimental scheme for cockroach allergen-induced mouse model of asthma. **(B)** Histological examination of mouse paraffin lung sections stained with hematoxylin and eosin (H&E). **(C, D)** Bronchoalveolar lavage (BAL) fluid total **(C)** and differential (eosinophil, macrophage, neutrophil, and lymphocyte, **(D)** cell counts as assessed by flow cytometry. **(E)** Serum levels of cockroach allergen-specific IgE and IgG1. **(F)** Levels of cytokines in BAL fluids. E-G, n = 6-11. Data represent means ± SEM. *p < 0.05, **p < 0.01, ***p < 0.001.

Environmental pollutant or allergen-activated NLRP3 inflammasome activation has been associated with exacerbation of asthma (43). Thus, we hypothesized that the ROS-activated NLRP3 inflammasome mediates allergen-induced airway inflammation. To test the hypothesis, we used MCC950, a highly selective NLRP3 inhibitor (59), to treat the CRE-induced mouse model following the protocol illustrated in **Figure 7A**. Consistent with our previous findings (55, 57), CRE sensitization and challenge induced increased airway inflammation. As expected, MCC950 treatment attenuated CRE-induced peribronchial inflammation (H&E, **Figure 7B**). These MCC950 treated mice also showed less total (**Figure 7C**) and differential inflammatory cells (**Figure 7D**) in the BAL fluids, lower levels of cockroach allergen-specific IgE and IgG1 in serum (**Figure 7E**), and IL-4, IL-5, and IL-13 production in BAL fluids (**Figure 7F**). These results indicate that inhibition of NLRP3 inflammasome alleviates cockroach allergen-induced airway inflammation.

### Inhibition of NLRP3 Inflammasome Suppresses Cockroach Allergen-Induced Muc5AC Expression

**Figure 8:**
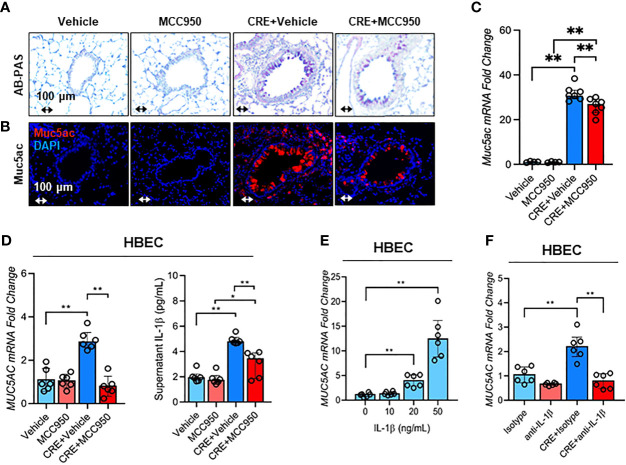
Inhibition of NLRP3 inflammasome suppresses cockroach allergen-induced MUS5AC expression. **(A)** Histological examination of mouse paraffin lung sections WITH AB-PAS staining. **(B)** Representative immunofluorescence images of Muc5ac expression in the lung tissues of CRE-induced mouse model of asthma with or without MCC950 treatment. **(C)** RT-PCR analysis of Muc5ac expression in the lung tissues of CRE-induced mouse model of asthma with or without MCC950 treatment (n = 6). **(D)** RT-PCR analysis of Muc5ac and ELISA analysis of IL-1β level in CRE-treated HBECs in the presence or absence of MCC950 (n = 6). **(E)** RT-PCR analysis of Muc5ac in HBECs treated with IL-1β at different concentrations (n = 6). **(F)**, RT-PCR analysis of Muc5ac in CRE-treated HBECs in the presence or absence of anti-IL-1β (n = 6). Data represent as medians with interquartile (IQR). *p < 0.05, **p < 0.01.

Consistent with a role for NLRP3 inflammasome in airway inflammation, we investigated whether NLRP3 inflammasome is involved in mediating CRE-induced Mucus production. Notably, MCC950 treatment inhibited CRE-induced mucus production as determined by AB-PAS staining (**Figure 8A**). The same pattern was noted for Muc5ac expression as evaluated by immunostaining (**Figure 8B**) and RT-PCR analysis of lung tissues (**Figure 8C**) of mouse model with or without MCC950 treatment. The regulation of NLRP3 on Muc5ac expression was further validated in HBECs by RT-PCR analysis. Similar to our previous observation, CRE induced Muc5ac overexpression, but which was significantly inhibited by MCC950 (**Figure 8D**). Consistently, IL-1β was increased in supernatants of CRE-treated HBECs, but reduced after treatment with MCC950. The increased IL-1β was also noted in the BAL fluids of CRE-treated mice, but reduced in mice treated with MCC950 (**Figure E5**). Given the significant changes for IL-1β, we hypothesized that the NLRP3 inflammasome-triggered IL-1β induces epithelial mucus production. Indeed, HBECs treated with IL-1β had increased expression of Muc5ac in a dose-dependent manner (**Figure 8E**). Furthermore, blockage of IL-1 β with anti- IL-1 β blocking antibody (2 µg/ml) inhibited the CRE-induced Muc5ac expression (**Figure 8F**), highlighting a significant role for IL-1β in mediating cockroach allergen-induced mucus production. Taken together, our results suggest that inhibition of NLRP3 inflammasome suppresses cockroach allergen-induced Muc5ac expression, which may be through controlling IL-1β secretion.

## Discussion

Mucus overproduction is a major risk factor for asthma morbidity and mortality (1). While evidence has shown that Muc5ac overexpression leads to the development of airway mucus plugging, AHR, and asthma exacerbations (7–10), the mechanisms underlying development and immune regulation of mucus production in response to allergens are not fully unveiled. In the current study, we found that cockroach allergen exposure can induce Muc5ac over expression and provided evidence that epithelial AhR is required in protecting against cockroach allergen-induced Muc5ac expression and airway inflammation. Furthermore, we identified a novel functional axis of ROS-NLRP3 inflammasome in mediating the AhR regulation on the Muc5ac expression and airway inflammation. These findings suggest that inhibition of ROS-NLRP3 inflammasome could provide a novel therapeutic strategy for mucus production and airway inflammation in asthma.

Muc5ac is the predominant mucin and mainly expressed in the upper airways, trachea, and bronchi, and tethering of Muc5ac to secreting club cells impairs mucus transport, consequently leading to the airway mucus plugging and progressive airway obstruction in asthma (1, 3, 7). Increased Muc5ac expression has been associated with airway obstruction, hyper-responsiveness, and severity of asthma (8, 9, 60, 61). Of interest, the increased Muc5ac concentration in induced sputum has been very recently identified as a major trigger in COPD initiation, progression, and exacerbation in a multicenter, observational study (11). However, the major risk factors driving the Muc5ac overexpression remain largely unknown. Recent studies demonstrated that genetic variations of Muc5ac (12, 62, 63), prolonged allergen exposure (64–66), and inflammatory mediators (67–69) are the key contributors to the Muc5ac overexpression. Of these, genetic variations of Muc5ac have been suggested to confer the susceptibility to environmental exposure and differential gene expression. On the other hand, environmental allergens [e.g., house dust mite (HDM) (64, 68), Cat allergen (66)] can induce Muc5ac overexpression. In the current study, we demonstrated that cockroach allergen exposure could induce Muc5ac overexpression by using both *in vitro* and *in vivo* analyses. While an increased expression of Muc5b, another major mucin produced from airway submucosal glands (70), was noted in the airways of asthma mouse model, no clear dose-dependent response was observed in the cockroach allergen-treated HBECs. Thus, Muc5ac, but not Muc5b, may be a major airway mucin selectively responding to cockroach allergen, highlighting that study targeting Muc5ac may have therapeutic potential in unplugging the airway mucus in asthma.

To investigate the molecular mechanisms underlying the cockroach allergen induced Muc5ac expression, we specifically focused on AhR signaling pathway. Our recent studies have demonstrated that AhR is critical in BaP co-exposure with Der f 1-induced IL-25, IL-33, and TSLP release from airway epithelial cells (32, 33). Furthermore, AhR signaling has been shown to regulate the BaP-induced Muc5ac overexpression in airway epithelial cells (34, 35). These findings led us to hypothesize that AhR in airway epithelial cells may regulate the cockroach allergen-induced Muc5ac expression. Although it is unclear how the AhR was activated, we found that cockroach allergen can induce AhR signaling activation in HBECs and mouse airways as evident by the increased expression of AhR and its downstream genes CYP1A1 and CYP1B1. Furthermore, our data in this and previous studies demonstrated that lack of AhR led to the increased allergic airway inflammation (58), suggesting that AhR is required in protecting against airway inflammation. This was also supported by the finding for Muc5ac. Mice with lack of AhR showed increased expression of Muc5ac, suggesting that AhR may inhibit Muc5ac expression and there might be a negative feedback loop for AhR and Muc5ac in response to cockroach allergen. This was consistent with the previous finding that a down-regulation of AhR was associated with increased Muc5ac in the lungs of HDM-induced mice (71). In contrast, active AhR can also induce Muc5ac expression and mucus production, such as AhR ligand BaP (34, 35), SARS-COV-2 (72). The reason for the opposite role of AhR in Muc5ac expression remains unclear, but it may be due to the AhR ligand-specificity or the AhR sequence and structure that are linked to the ligand-activation or -inhibition, which would be of interest for further studies. Taken together, our data suggest that epithelial AhR plays a protective role in cockroach allergen-induced airway inflammation and mucus production.

NLRP3 inflammasome is an intracellular sensor that detects environmental irritants and endogenous danger signals and has been implicated to be an essential player in the pathogenesis of human metabolic and auto-inflammatory diseases (36–39). While NLRP3 inflammasome has been initially recognized to provide protective immunity by facilitating the clearance of pathogens in the airways, over-activation of NLRP3 inflammasome can lead to the exacerbation of airway inflammation and asthma (43, 73). Of interest, NLRP3 inflammasome activation has been linked with Muc5ac overproduction (37, 44, 45, 74) and allergic airway inflammation (42, 46–49, 74). These findings raise the possibility that allergen-induced NLRP3 inflammasome activation mediates the AhR signaling pathway-regulated airway inflammation and Muc5ac expression. Indeed, both RT-PCR and western blot (data not shown) analyses demonstrated that expression of NLRP3 and its related genes were significantly increased in HBECs after exposure to cockroach allergen, supporting the notion that cockroach allergen exposure can induce NLRP3 inflammasome activation. Next, we tested whether AhR regulates cockroach allergen-induced NLRP3 inflammasome activation. In fact, AhR has been reported to inhibit NLRP3 transcription and consequent activation of NLRP3 inflammasome in peritoneal macrophages through binding to the xenobiotic response element (XRE) in the NLRP3 promoter (75). Further evidence indicated that AhR is an inhibitor of NLRP3 inflammasome in bowel inflammation and colitis-associated colorectal cancer (76). In this study, we demonstrated a markedly increased expression of NLRP3 and its related genes in response to cockroach allergen by *in vitro* and *in vivo* analyses, suggesting that AhR situated in the airways inhibits cockroach allergen-induced NLRP3 inflammasome activation. Although pro-inflammatory IL-1β and IL-18 were increased in response to cockroach allergen, IL-1β, but not IL-18, was further enhanced by the deficiency of AhR, highlighting that IL-1β, but not IL-18, is a major AhR-mediated inflammasome downstream pro-inflammatory cytokine.

It has been documented that excessive generation of ROS observed in the airways of asthma patients causes oxidative stress and chronic inflammatory responses in asthma (29, 54, 77–79) and ROS are crucial for NLRP3 inflammasome activation in bronchial epithelial cells (39, 46, 50). It is possible that AhR signaling controls NLRP3 inflammasome activation through modulating ROS generation. Indeed, we have previously shown that AhR signaling regulates ROS generation that contributes to mast cell degranulation and activation (30, 31) and epithelial cytokine release (32). Thus, we examined whether AhR regulates cockroach allergen-induced airway epithelial ROS and determined whether ROS is required in cockroach allergen-induced NLRP3 inflammasome activation. We found that cockroach allergen-induced intracellular ROS were enhanced in HBECs either with AhR knockdown (data not shown) or pre-treated with AhR antagonist CH223191. The results were further confirmed by the *in vivo* analyses of ROS production in the lung tissues of CRE-treated mice. Thus, our data support that AhR protects against cockroach allergen-induced epithelial ROS, particularly mitochondrial ROS production. However, the exact mechanisms underlying the AhR-involved regulation of ROS remains unclear, thus warranting further, in-depth investigations in the future.

Further studies examined whether ROS play a role in the cockroach allergen-induced NLRP3 inflammasome activation. Several studies have provided evidence that ROS are closely associated with NLRP3 inflammasome activation (39, 46, 50). Particularly, mitochondrial ROS have been considered as a major cellular source to induce NLRP3 inflammasome activation (80). Consistent with these findings, we found that cockroach allergen-induced NLRP3 inflammasome activation in HBECs was suppressed by antioxidant NAC or mitochondrial-targeted antioxidant MitoTEMPO, highlighting that ROS are crucial for cockroach allergen-induced NLRP3 inflammasome activation. Thus, our data support the notion that that AhR regulates NLRP3 inflammasome activation through controlling cockroach allergen-induced ROS, particularly mitochondrial ROS generation.

Growing evidence has suggested a major role for NLRP3 inflammasome activation in the development of airway inflammation (41, 42) and exacerbation of asthma features (43). Using mice deficient in NLRP3 and IL-1β, studies have documented reduced airway inflammation, eosinophil influx, suppressed mucus secretion, dampened airway hyper-responsiveness, and cytokine release in OVA-challenged mice, highlighting the implication of NLRP3 activation in airway inflammation (49, 81). In the present study, we investigated whether the ROS-activated NLRP3 inflammasome mediates allergen-induced airway inflammation and mucus overproduction by using MCC950, a highly selective NLRP3 inhibitor. As expected, MCC950 treatment abrogated CRE-induced airway inflammation, eosinophil influx, IgE levels, and Th2-associated cytokines. Consistently, MCC950 treatment also inhibited CRE-induced Muc5ac expression, highlighting the significance of NLRP3 inflammasome activation in mucus production (37, 44, 45). This finding was further supported by a recent study showring that inhibition of the NLRP3 inflammasome with MCC950 attenuated HDM-induced allergic inflammation and mucus production (74). Furthermore, treatment with IL-1β led to a dose-dependent response for Muc5ac expression, and blockade of IL-1β with neutralizing antibody significantly prevented Muc5ac expression in HBECs. Of interest, IL-1β signaling has been shown to be critical in inducing neutrophil chemotactic factors, IL-33, and Muc5ac expression at viral-induced asthma exacerbation (82). These data suggest that blockade of NLRP3 inflammasome and IL-1β could provide new treatment options for asthma.

Taken together, we provided evidence that cockroach allergen exposure can induce MUC5AC over expression and epithelial AhR protects against cockroach allergen-induced MUC5AC expression and airway inflammation. Further studies suggest that cockroach allergen can induce NLRP3 inflammasome activation and epithelial AhR prevents cockroach allergen-induced NLRP3 inflammasome activation. Mechanistically, our studies suggest a novel mechanism that AhR regulates NLRP3 inflammasome activation through controlling cockroach allergen-induced mitochondrial ROS. Mitochondrial ROS are a major cellular source to induce NLRP3 inflammasome activation. Furthermore, we identified a new player, the NLRP3 inflammasome-mediated IL-1β, which can drive airway mucus overproduction in asthma (See Graphic abstract). One of the major limitations is the lack of primary human bronchial epithelial cells that could be used for this study. Instead, HBECs (HBEC3-KT, CRL-4051), an immortalized cell line under submerged cultures, were selected for this study, which may not perfectly reflect the real allergen triggered changes of Muc5ac. Thus, future studies will include other epithelial cell lines, such as the primary bronchial epithelial cells PCS-300-010, under air-liquid interface culture, to further study the regulation of Muc5ac expression in the differentiated mucus-secreting goblet-like cells (83). Collectively, these studies highlight a critical role for the activated AhR signaling in the airway epithelial cells in protecting allergic airway inflammation and mucus production, and suggest a novel therapeutic strategy targeting the functional axis of ROS-NLRP3 inflammasome in patients with allergic asthma.

## Data Availability Statement

The original contributions presented in the study are included in the article/**Supplementary Material**. Further inquiries can be directed to the corresponding authors.

## Ethics Statement

The animal study was reviewed and approved by Johns Hopkins.

## Author Contributions

XH, YS, YZ, JW, XZ, WT, and WK performed experiments, analyzed data, and review the manuscript. XH and PG wrote the manuscript. JF and PG designed and supervised the study, and wrote the manuscript. All authors contributed to the article and approved the submitted version.

## Funding

This work was supported by grants from the US National Institutes of Health (NIH) (1R56AI143668, R21 AI137547, 1R01AI153331, and R01AI141642 to PG) and the National Natural Science Foundation of China (81873407 to JF).

## Conflict of Interest

The authors declare that the research was conducted in the absence of any commercial or financial relationships that could be construed as a potential conflict of interest.

## Publisher’s Note

All claims expressed in this article are solely those of the authors and do not necessarily represent those of their affiliated organizations, or those of the publisher, the editors and the reviewers. Any product that may be evaluated in this article, or claim that may be made by its manufacturer, is not guaranteed or endorsed by the publisher.

## Abbreviations

CRE, Cockroach extract; AhR, Aryl hydrocarbon receptor; HBEC, Human bronchial epithelial cell; ROS, Reactive oxidative stress; WT, Wild-type; mtROS, Mitochondrial reactive oxidative stress; NAC, N-Acetyl Cysteine; BAL, Bronchoalveolar lavage; NLRP3, Nucleotide-binding oligomerization domain (NOD)-like receptor; family pyrin domain-containing 3; ASC, Apoptotic speck protein containing a caspase recruitment domain; HDM, House dust mite; XRE, Xenobiotic response element; PAS, Alcian Blue periodic acid-Schiff; RT-PCR, Quantitative real-time PCR; FBS, Fetal bovine serum; H&E, Hematoxylin and eosin; PAS, Alcian Blue periodic acid-Schiff.
