# Supplementary figures and images for: Epithelial Aryl Hydrocarbon Receptor Protects From Mucus Production by Inhibiting ROS-Triggered NLRP3 Inflammasome in Asthma
Source: Front Immunol. 2021 Nov 15;12:767508. doi: 10.3389/fimmu.2021.767508 (PMC8634667; doi:10.3389/fimmu.2021.767508)

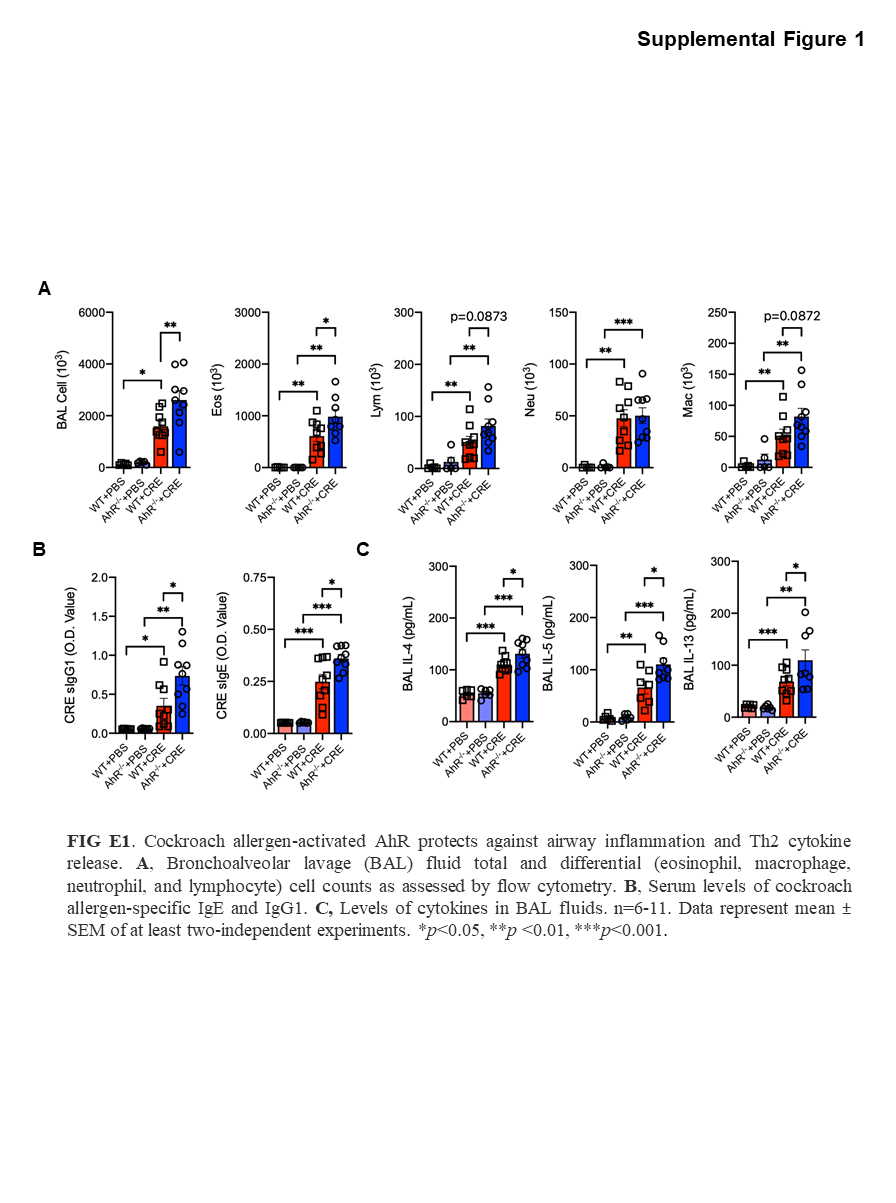

Supplement: Supplementary file 1 [file Image_1.tif]

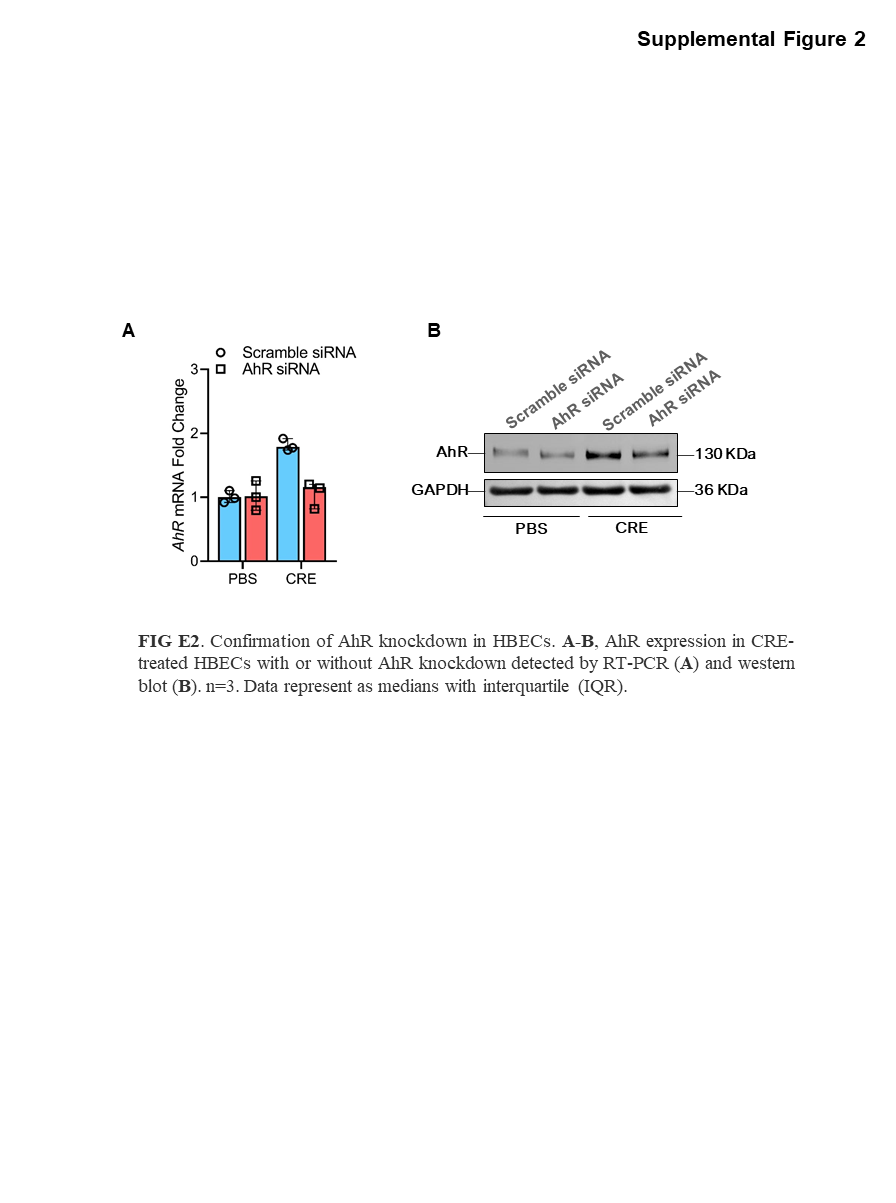

Supplement: Supplementary file 2 [file Image_2.tif]

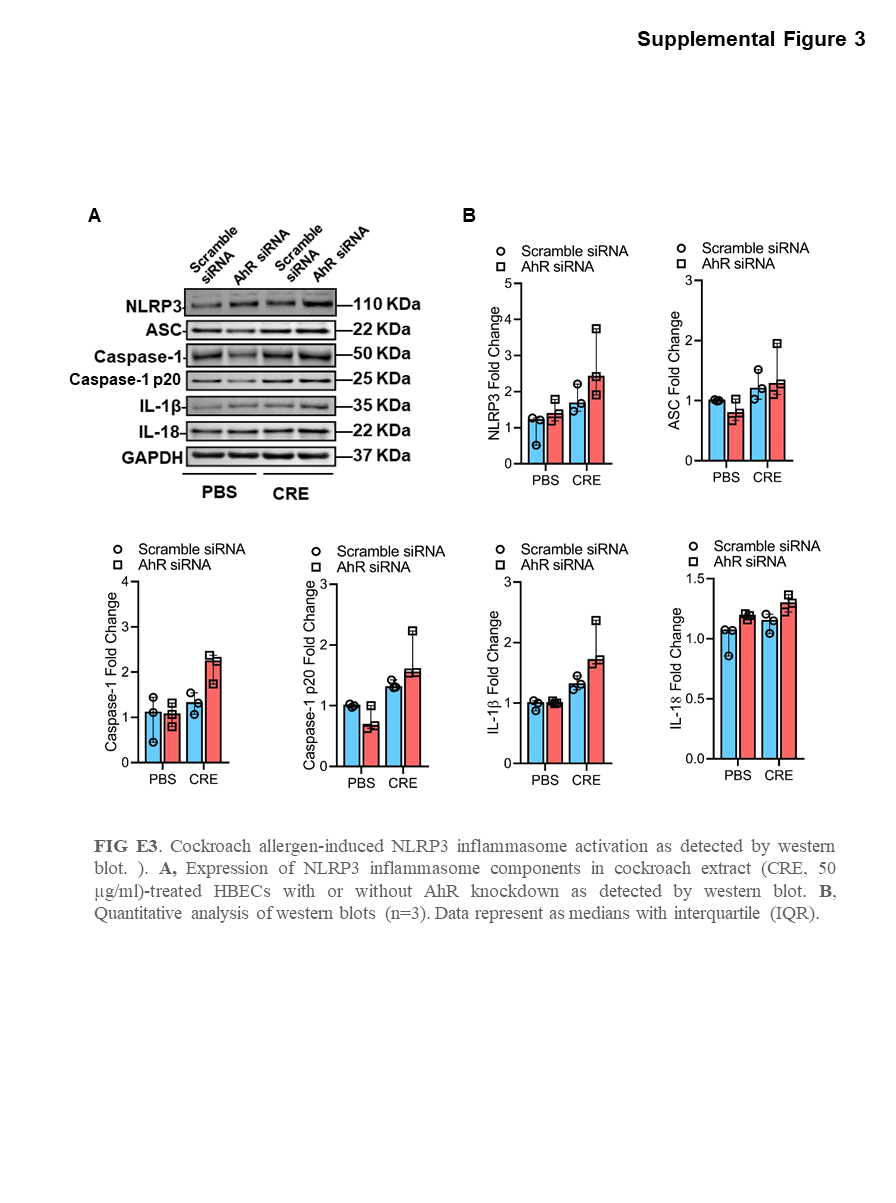

Supplement: Supplementary file 3 [file Image_3.tif]

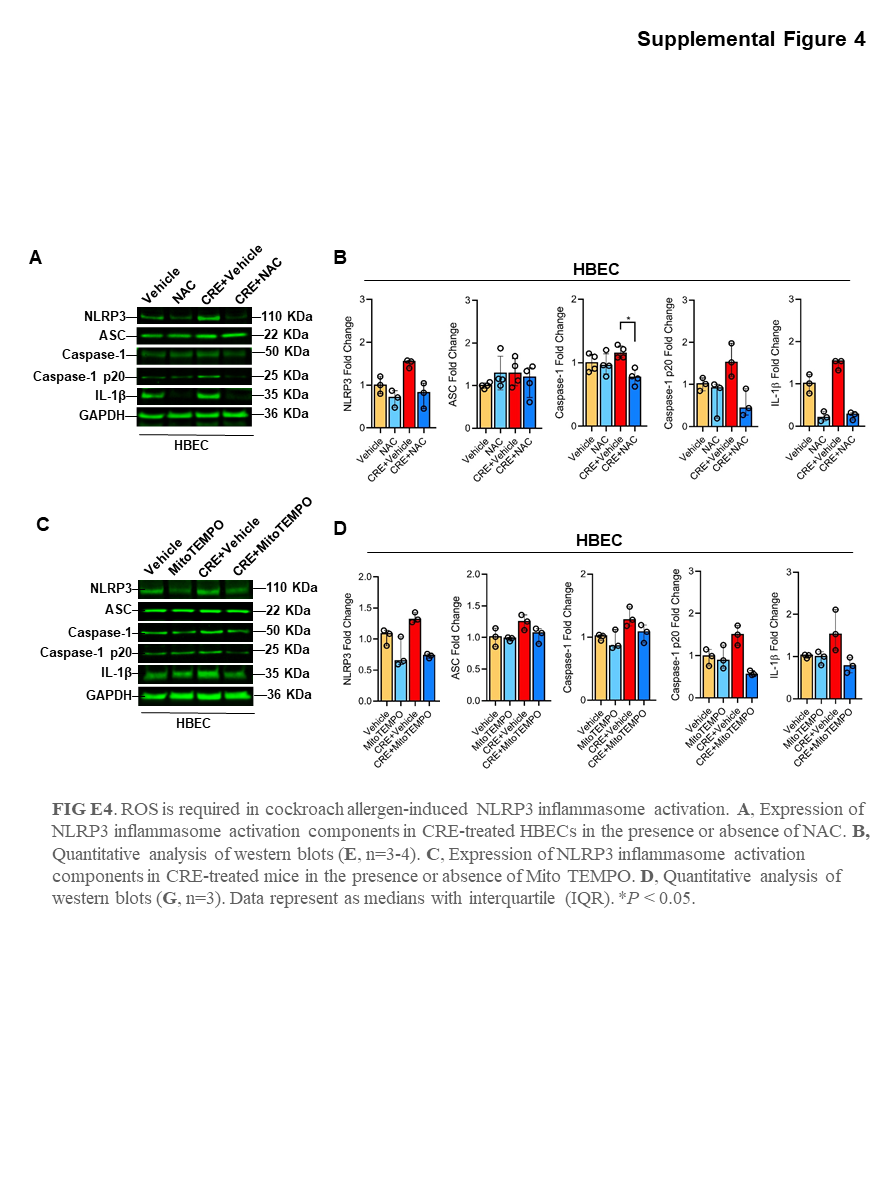

Supplement: Supplementary file 4 [file Image_4.tif]

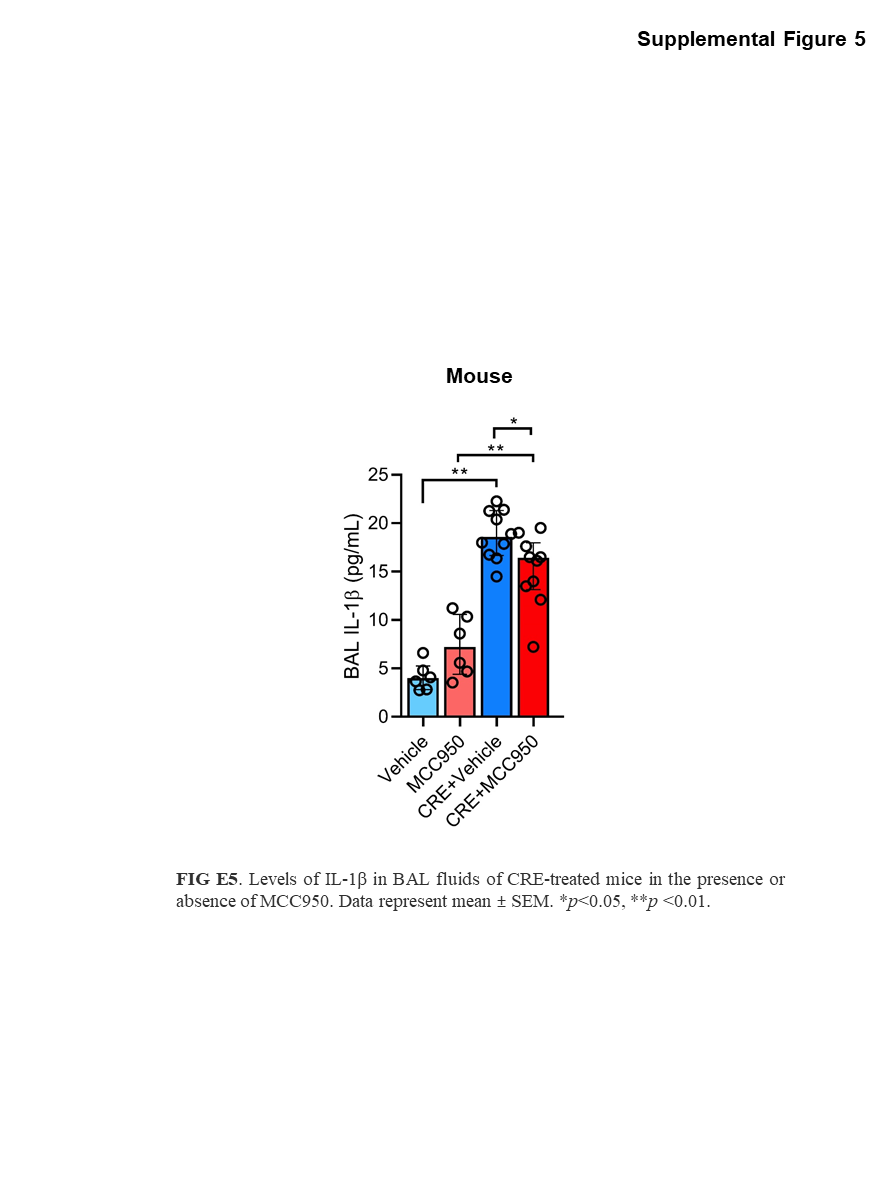

Supplement: Supplementary file 5 [file Image_5.tif]
